# Impact of Ni Content on the Structure and Sonophotocatalytic Activity of Ni-Zn-Co Ferrite Nanoparticles

**DOI:** 10.3390/ijms232214167

**Published:** 2022-11-16

**Authors:** Thomas Dippong, Oana Cadar, Firuta Goga, Dana Toloman, Erika Andrea Levei

**Affiliations:** 1 Faculty of Science, Technical University of Cluj-Napoca, 76 Victoriei Street, 430122 Baia Mare, Romania; 2 Research Institute for Analytical Instrumentation Subsidiary, National Institute for Research and Development for Optoelectronics INOE 2000, 67 Donath Street, 400293 Cluj-Napoca, Romania; 3 Faculty of Chemistry and Chemical Engineering, Babes-Bolyai University, 11 Arany Janos Street, 400028 Cluj-Napoca, Romania; 4 National Institute for Research and Development of Isotopic and Molecular Technologies, 67-103 Donath Street, 400293 Cluj-Napoca, Romania

**Keywords:** nickel-zinc-cobalt ferrite, thermal behavior, crystalline phase, sonophotocatalysis

## Abstract

The structure, morphology, and sonophotocatalytic activity of Ni-Zn-Co ferrite nanoparticles, embedded in a SiO_2_ matrix and produced by a modified sol-gel method, followed by thermal treatment, were investigated. The thermal analysis confirmed the formation of metal succinate precursors up to 200 °C, their decomposition to metal oxides and the formation of Ni-Zn-Co ferrites up to 500 °C. The crystalline phases, crystallite size and lattice parameter were determined based on X-ray diffraction patterns. Transmission electron microscopy revealed the shape, size, and distribution pattern of the ferrite nanoparticles. The particle sizes ranged between 34 and 40 nm. All the samples showed optical responses in the visible range. The best sonophotocatalytic activity against the rhodamine B solution under visible irradiation was obtained for Ni_0.3_Zn_0.3_Co_0.4_Fe_2_O_4_@SiO_2_.

## 1. Introduction

Despite the measures taken to reduce pollution, industrial effluents containing dyes and pigments used in the textile industry often resurface in the surrounding environment. Dyes are complex organic structures with a high resistance to chemical and biological degradation, high water solubility, and have a negative impact on the environment, particularly aquatic ecosystems [1,2]. Therefore, the efficient treatment of industrial effluents and wastewaters containing dyes is crucial for environmental protection.

The photocatalytic degradation of dyes is a simple, cost-effective, and environmentally friendly approach for wastewater treatment as it allows the decomposition of complex organic structures into CO_2_ and water [3,4]. In the last few years, the use of sonophotocatalysis for the degradation of a wide range of organic pollutants in aqueous systems has been the topic of several studies [5,6]. Sonophotocatalysis use the synergistic effects of ultrasonic waves, UV-Vis irradiation and photocatalyst to form highly reactive free radicals in an aqueous medium that further react with dyes and lead to their degradation [7]. By providing additional nuclei, the heterogeneous catalyst enhances the formation of cavitation bubbles, which in turn increases the formation of reactive radicals through water pyrolysis [5]. The mechanism of sonophotocatalytic degradation, as well as the main advantages of combined ultrasound and photocatalytic processes, are presented by Abdurahman et al. [5]. The high energy consumption of sonophotocatalysis makes its large-scale application difficult, however, the high costs could be compensated by the low time required for the degradation of organic compounds [7]. Due to their magnetic properties, their recovery using an external magnetic field and their reuse is possible [8,9].

Oxides containing at least two types of metals are potential candidates for photoelectrochemistry and photocatalysis due to their band structure and energy position [10]. The bandgap of several MFe_2_O_4_-type ferrites are presented by Dillert et al. [11]. Nanosized spinel-type ferrites, containing first-row transition metals such are Ni, Co, Zn, Mn are attractive materials in electronics, magnetic storage, ferrofluid technology, gas sensors, catalysis, photocatalysis and biomedicine, including magnet-guided drug carriers, contrast agents and tracers for positive magnetic resonance imaging [12,13,14,15,16,17]. They are also promising candidates for wastewater treatment as they could act both as adsorbents, due to their high specific surface area, and as photocatalysis, due to their low energy band gap, that allow the conversion of UV or visible light into chemical energy that favors the degradation of dyes [8,17,18,19,20,21]. The strong photodegradation capacity of 3d transition metal ferrites such as CoFe_2_O_4_, CuFe_2_O_4_, NiFe_2_O_4_, and ZnFe_2_O_4_, with magnetic properties, was previously demonstrated for different organic compounds [12,13,21,22,23,24,25,26,27]. The surface coating of the ferrite nanoparticles with different materials, especially semiconductor materials such as TiO_2_ and SiO_2_, was proven to enhance the photocatalytic activity [28]. CoFe_2_O_4_ nanoparticles coated with TiO_2_–SiO_2_ efficiently degraded (up to 98%) the methylene blue dye [2], while Rhodamine B (RhB) degradation by CoFe_2_O_4_, was only 80% [29,30]. The enhancement of the photocatalytic performance of Ni_x_Co_1-x_Fe_2_O_4_, prepared by the coprecipitation method against methylene blue at a high Ni content, was reported by Lassoued and Li [31]. A good photodegradation efficiency (about 80%) of methylene blue under visible light irradiation was also delineated for Co-Zn ferrite with various Co and Zn contents, obtained using the citrate precursor method [32]. The increase of the rate constant with the increasing Co content was also observed [32]. The photocatalytic activity of Co_0.6_Zn_0.4_Ni_x_Fe_2-x_O_4_ powders, obtained by the sol-gel method under visible light against methyl orange dye in an aqueous solution, was also reported [33].

The spinel ferrite properties are determined by their composition, structure, particle size, and morphology [34,35,36,37]. These characteristics strongly depend on the synthesis route, chemical composition, doping cations, and sintering conditions [36,38]. The change in preparation method and temperature affects the microstructure, cation distribution among tetrahedral (A) and octahedral (B) sites and magnetic properties. Thus, to produce spinel ferrite nanoparticles with a desired stoichiometry, high compositional control, excellent chemical stability, high purity, crystallinity, saturation magnetization, and low coercivity are of interest and the selection of the synthesis route is critical [15,38].

Soft chemical routes such as sol-gel, solid-phase, hydrothermal, coprecipitation, sonochemical, spray pyrolysis, citrate gel, microwave refluxing, flash auto combustion, etc., are currently preferred for the synthesis of nano ferrites [17,37,39,40]. The solid-state reaction is a simple and attractive preparation method that allows large productivity and well-controllable grain sizes [41]. The conventional ceramic method is based on the solid-state reaction of metal oxides/carbonates at high temperatures (>1000 °C) and produces particles in the micrometer regime; however, agglomeration due to slow reaction kinetics is unavoidable [39]. Hence, wet chemical methods have been used intensively to avoid the limitations of conventional ceramic methods and to produce nanoscale materials with improved magnetic properties [35]. Wet chemical routes, such as hydrothermal, sol-gel method, and auto combustion, have been employed to obtain ferrite nanoparticles at low temperatures [34,39,41]. The main drawback of the wet methods is the formation of different oxide impurities, particularly Fe_2_O_3_ [17]. Highly homogenous Ni-Zn nanoferrite powders can be easily produced by a wet chemical route, using low-cost raw materials, and in air atmospheres. Its properties can be adjusted to fit the requirements of different applications by appropriately adjusting the Ni-Zn ratio and the sintering process [37,42]. The properties of Ni-Zn nanoferrites can be further improved by adding low amounts of other divalent ions such as Co^2+^ [37].

This paper investigates the formation, structure, morphology, and sonophotocatalytic activity of mixed Ni-Zn-Co ferrites embedded in SiO_2_, obtained by a modified sol-gel method and followed by thermal treatment. The reaction progress was investigated through thermal analysis (TG-DTA) and Fourier transform infrared spectroscopy (FT-IR), while the Ni-Zn-Co ferrites composition was investigated by inductively coupled plasma optical emission spectrometry (ICP-OES). The formation of the crystalline phase, crystallite size, and lattice constant were monitored by X-ray diffraction (XRD). The surface (specific surface area and porosity) was investigated using the Brunauer-Emmett-Teller (BET) method. The sonophotocatalytic properties of the samples were evaluated under visible light irradiation, assisted by sonication against RhB.

## 2. Results and Discussion

### 2.1. Thermal Analysis

**Figure 1:**
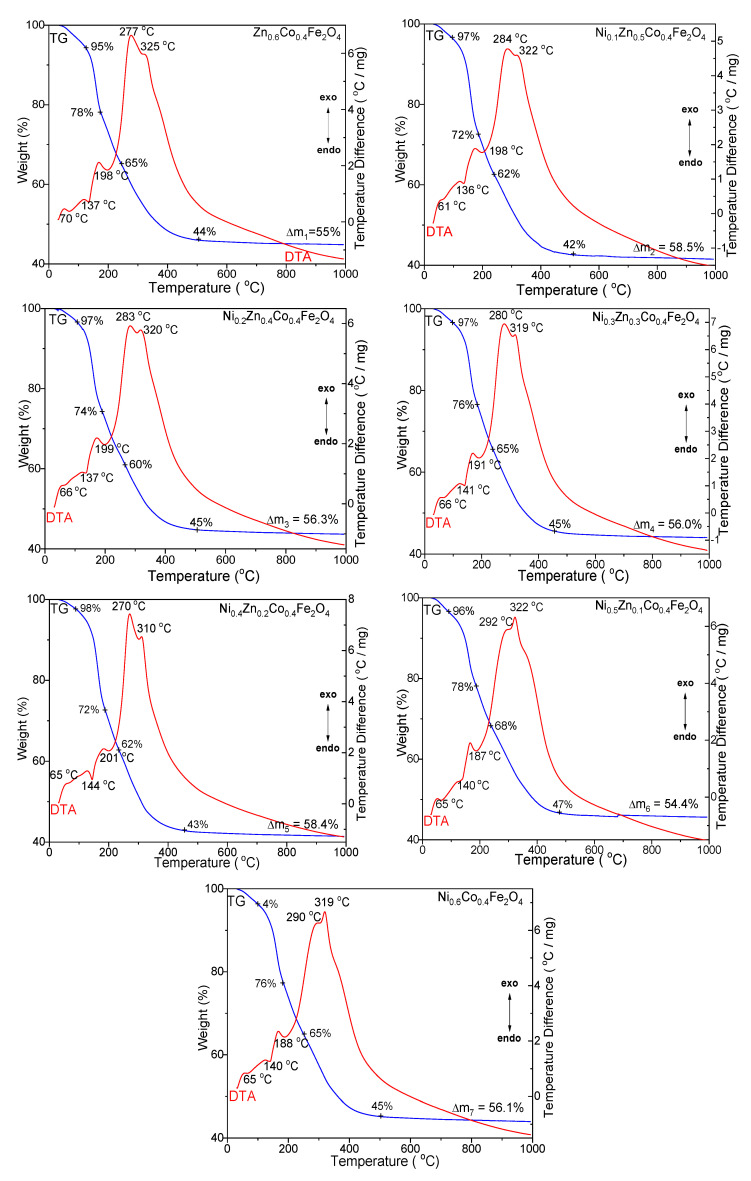
TG-DTA diagrams of Zn_0.6_Co_0.4_Fe_2_O_4_@SiO_2_, Ni_0.1_Zn_0.5_Co_0.4_Fe_2_O_4_@SiO_2_, Ni_0.2_Zn_0.4_Co_0.4_Fe_2_O_4_@SiO_2_, Ni_0.3_Zn_0.3_Co_0.4_Fe_2_O_4_@SiO_2_, Ni_0.4_Zn_0.2_Co_0.4_Fe_2_O_4_@SiO_2_, Ni_0.5_Zn_0.1_Co_0.4_Fe_2_O_4_@SiO_2_ and Ni_0.6_Co_0.4_Fe_2_O_4_@SiO_2_ samples.

The TG-DTA curves of all of the samples show three endothermic and two exothermic processes characterized by very close, overlapping peaks (Figure 1).

The endothermic effect at 61–70 °C, accompanied by 3–5% mass loss, is attributed to the loss of crystallization and constitution water. The endothermic effect at 136–144 °C, accompanied by 17–26% mass loss, is assigned to the formation of divalent metal precursors (Co, Ni, and Zn succinates), while the endothermic effect at 187–201 °C accompanied by 9–14% mass loss, is ascribed to the formation of a trivalent metal precursor (Fe succinate). The distinct behavior of Fe succinate, compared to the divalent metal (Co, Ni, Zn) succinates, can be attributed to the redox reaction between Fe(NO_3_)_3_ and 1,4BD, as well as to the stronger acidity of the aqua-cation [Fe(H_2_O)_6_]_3_ [43]. The overlapping exothermic effects, at 270–292 °C and 310–325 °C, accompanied by 19–25% mass loss, are attributed to the decomposition of metal succinates to metal oxides, which leads to the formation of ferrites.

The exothermic peak, characteristic of the decomposition of divalent metal succinates, decreases with the increasing Zn content and shifts toward higher temperatures, leading to the increase of the exothermic peak, attributed to the decomposition of the Fe succinates. The SiO_2_ matrix suffers various transformations during the thermal process, making the demarcation of the processes attributed to the formation and decomposition of succinate precursors difficult [44]. The total mass loss increases in the following order: Ni_0.5_Zn_0.1_Co_0.4_Fe_2_O_4_@SiO_2_ < Zn_0.6_Co_0.4_Fe_2_O_4_@SiO_2_ < Ni_0.3_Zn_0.3_Co_0.4_Fe_2_O_4_@SiO_2_ < Ni_0.6_Co_0.4_Fe_2_O_4_@SiO_2_ < Ni_0.2_Zn_0.4_Co_0.4_Fe_2_O_4_@SiO_2_ < Ni_0.4_Zn_0.2_Co_0.4_Fe_2_O_4_@SiO_2_ < Ni_0.1_Zn_0.5_Co_0.4_Fe_2_O_4_@SiO_2_.

### 2.2. FT-IR Analysis

**Figure 2:**
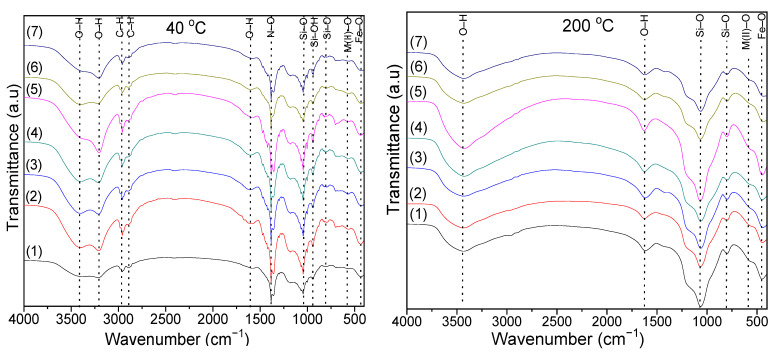
FT-IR spectra of Zn_0.6_Co_0.4_Fe_2_O_4_@SiO_2_ (1) Ni_0.1_Zn_0.5_Co_0.4_Fe_2_O_4_@SiO_2_ (2), Ni_0.2_Zn_0.4_Co_0.4_Fe_2_O_4_@SiO_2_ (3), Ni_0.3_Zn_0.3_Co_0.4_Fe_2_O_4_@SiO_2_ (4) Ni_0.4_Zn_0.2_Co_0.4_Fe_2_O_4_@SiO_2_ (5) Ni_0.5_Zn_0.1_Co_0.4_Fe_2_O_4_@SiO_2_ (6) and Ni_0.6_Co_0.4_Fe_2_O_4_@SiO_2_ (7) samples at 40 and 200 °C.

As vibrational modes in FT-IR spectroscopy are determined by the bond type, the symmetry of the lattice sites and the elements in the crystal lattice, the monitoring of the ferrite formation process is possible [44]. The FT-IR spectra of the gels dried at 40 and 200 °C are presented in Figure 2.

**Figure 3:**
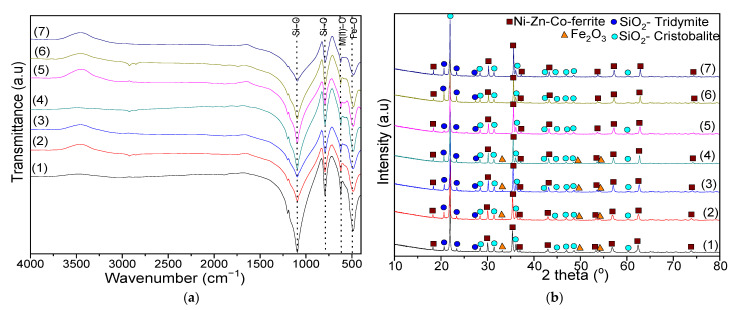
FT-IR spectra (**a**) and XRD patterns (**b**) of Zn_0.6_Co_0.4_Fe_2_O_4_@SiO_2_ (1), Ni_0.1_Zn_0.5_Co_0.4_Fe_2_O_4_@SiO_2_ (2), Ni_0.2_Zn_0.4_Co_0.4_Fe_2_O_4_@SiO_2_ (3), Ni_0.3_Zn_0.3_Co_0.4_Fe_2_O_4_@SiO_2_ (4), Ni_0.4_Zn_0.2_Co_0.4_Fe_2_O_4_@SiO_2_ (5), Ni_0.5_Zn_0.1_Co_0.4_Fe_2_O_4_@SiO_2_ (6) and Ni_0.6_Co_0.4_Fe_2_O_4_@SiO_2_ (7) samples at 1000 °C.

In the case of the samples dried at 40 °C, the intense band at 1379–1389 cm^−1^ is associated with the N-O bonds stretching vibration in metal nitrates. This band disappears in the case of samples dried at 200 °C, indicating the decomposition of nitrates [44]. The bands at 2958–2963 cm^−1^ and 2872–2888 cm^−1^ are specific to the symmetric and asymmetric vibration of C-H bonds in 1,4-BD or succinate precursors. These bands also disappear in samples heat-treated at 200 °C. The bands at 1578–1605 and 3200–3210 cm^−1^ are attributed to the stretching and bending vibrations of O-H in 1,4-BD and adsorbed molecular water [44,45]. In the samples dried at 200 °C, the band at 3200–3210 cm^−1^ is shifted towards higher wavenumbers (3421–3437 cm^−1^), indicating that the metal succinates are hygroscopic [44,45]. The presence of this absorption band could also be due to the O-H stretching vibration and Si-OH deformation vibration caused by the hydrolysis of –Si(OC_2_H_5_)_4_ [44,45]. For samples dried at 40 and 200 °C, the bands at 557–568 cm^−1^ are attributed to Ni-O and Zn-O vibrations, while the band at 433–452 cm^−1^ is attributed to the Fe-O vibration in the nitrates [44,45]. In samples at 40 °C, the band at 683–393 cm^−1^ is assigned to the Co-O bond vibration in the cobalt nitrate [44,45]. The formation of the SiO_2_ matrix in the samples dried at 40 and 200 °C is confirmed by the presence of specific bands of Si-O bond vibration (433–452 cm^−1^), cyclic Si-O-Si bonds vibration (557–568 cm^−1^, more noticeable in samples dried at 40 °C), Si-O symmetric stretching and bending vibration (792–815 cm^−1^), Si-OH bonds (938–943 cm^−1^, well delimited only in case of samples dried at 40 °C) vibration and Si-O-Si bonds stretching vibration (1045 cm^−1^ at 40 °C and 1058–1068 cm^−1^ at 200 °C). The band at 938–943 cm^−1^ is distinguishable for the samples dried at 40 °C, indicating the presence of unreacted TEOS, while the band at 1045 cm^−1^ suggests the formation of amorphous SiO_2_ [44,45]. Figure 3a shows the FT-IR spectra of NCs thermally treated at 1000 °C. The band at 618–626 cm^−1^ is attributed to the vibration of the M(II)-O (Co-O, Ni-O, Zn-O) bonds, while that at 485–490 cm^−1^ is attributed to the Fe-O bond [44,45,46]. 

In comparison to the samples dried at 40 °C and 200 °C, in the samples thermally treated at 1000 °C, the wavenumbers specific to Co-O bond vibration decrease, and the wavenumbers specific to M(II)-O increase. The Jan-Teller effect, determined by the presence of Fe^2+^ ions in the sublattices, can lead to band splitting, small bands and/or shoulders [47]. The Fe^2+^ ions may result from the hopping process, namely M^2+^ + Fe^3+^ ↔ M^3+^ + Fe^2+^ (M = Co, Ni, Zn). The Co^3+^ ion may migrate to tetrahedral (A) sites, while the Fe^2+^ ions remain in their sites [31].

The bands at 790–795 cm^−1^ are characteristic for the vibration of the Si-O bond in SiO_2_ matrix, while those at 1090–1095 cm^−1^ and 485–490 cm^−1^ are characteristic to the stretching and bending vibration of Si-O-Si chains and show a low degree of polycondensation of the SiO_2_ network [44,45]. The difference in band position could be attributed to the difference in M-O distance in the tetrahedral and octahedral sites [39].

### 2.3. XRD Analysis

The XRD patterns (Figure 3b) confirm the presence of well-crystallized ferrites in all of the samples, while the positions and intensities of the diffraction lines support the spinel structure [47]. The peaks with 2θ values of 30.07, 35.42, 37.07, 43.05, 53.41, 56.94, and 62.52 correspond to the (220), (311), (222), (400), (422), (511), and (440) planes [41,48]. In all of the samples, the local crystal structure is cubic spinel-type, belonging to the *Fd-3m* space group [21,40,41]. Additionally, two crystalline phases of the SiO_2_ matrix (SiO_2_-cristobalite, JCPDS card 39-1425 [48] and SiO_2_-tridymite, JCPDS card 042-1401 [48]) are identified. In the case of Zn_0.6_Co_0.4_Fe_2_O_4_@SiO_2_, the well-crystallized spinel is composed by CoFe_2_O_4_ (JCPDS card 22-1086 [48]) and ZnFe_2_O_4_ (JCPDS card 70-6491 [48]. In the case of Ni_0.6_Co_0.4_Fe_2_O_4_@SiO_2_, the well-crystallized spinel is composed of CoFe_2_O_4_ and NiFe_2_O_4_ (JCPDS card 74-2081 [48]), while in the other samples (Ni_0.1_Zn_0.5_Co_0.4_Fe_2_O_4_@SiO_2_, Ni_0.2_Zn_0.4_Co_0.4_Fe_2_O_4_@SiO_2_, Ni_0.3_Zn_0.3_Co_0.4_Fe_2_O_4_@SiO_2_, Ni_0.4_Zn_0.2_Co_0.4_Fe_2_O_4_@SiO_2_, and Ni_0.5_Zn_0.1_Co_0.4_Fe_2_O_4_@SiO_2_), the crystalline phase contains NiFe_2_O_4_, ZnFe_2_O_4_ and CoFe_2_O_4_. In ferrites with a high Zn content, the presence of hematite (Fe_2_O_3_, JCPDS card 89-0599 [48]) is also remarked. The presence of Fe_2_O_3_ indicates the decomposition of Fe(NO_3_)_3_ into Fe_2_O_3_, leading to the formation of spinel ferrite [39]. The excess of metal oxides in insoluble secondary phases (Fe_2_O_3_) can contribute to the densification by generating high pore volumes and demagnetizing fields. During synthesis, the homogeneity of the metal oxide particles may result in higher defects and pore volumes in the final products [36,42]. 

The average crystallites size (D_XRD_) was calculated using the Scherrer equation (Equation (1)).

(1) $$D_{\mathrm{XRD}}=\frac{0.9\;\cdot \lambda \;}{\beta \;\cdot \cos\;\theta }$$

where λ is the wavelength of the CuK_α_ radiation (1.5406 Å), β is the full width at half-maximum intensity (FWHM), hkl are the Miller indices and θ is the Bragg angle (°) [43,44,49,50].

The lattice constant (a), was calculated from Bragg’s law with Nelson-Riley Equation (2) [44,50].

(2) $$a=\frac{\lambda \sqrt{h^{2}+k^{2}+l^{2}}}{2\cdot \sin\theta }$$

where λ is the wavelength of CuK_α_ radiation (1.5406 Å), hkl are the Miller indices, θ is the Bragg angle (°) [44,50].

The unit cell volume (V) and the hopping length (L) of magnetic ions for tetrahedral (A) and octahedral (B) sites were calculated using Equations (3)–(5) [44,50].

(3) $$V=a^{3}$$

(4) $$L_{A}=0.25\cdot a\sqrt{3}$$

(5) $$L_{B}=0.25\cdot a\sqrt{2}$$

where a is the lattice constant (Å).

The average crystallite size lies in the nanocrystalline range and increases with the increasing Ni content, while the lattice parameter (a) decreases with the increasing Ni content (Table 1). 

The change in the lattice constant (a) generates internal stress and suppresses additional grain growth during thermal treatment [44,50,51]. The tetrahedral (A) sites have smaller radii (0.52 Å) than the octahedral (B) site (0.81 Å) [9]. The ionic radii of Ni^2+^ (0.69 Å), Zn^2+^ (0.74 Å) and Co^2+^ (0.75 Å) ions are larger than the ionic radius of Fe^3+^ (0.64 Å) [3,38,52]. The amorphous to crystalline phase transformation and the relative content of crystalline phases, after thermal treatment at 1000 °C, were assessed using the relative degree of crystallinity (DC), calculated as the ratio between the area of diffraction peaks and the total area of diffraction peaks and halos. The DC increases with the increase of the crystallite size and Ni content. The Reference Intensity Ratio (RIR) method was used for the quantitative phase analysis of NCs thermally treated at 1000 °C.

### 2.4. Elemental Analysis

The Ni/Zn/Co molar ratios, determined by microwave digestion and combined with inductively coupled plasma optical emission spectrometry, are in good agreement with the theoretical values (Table 1).

### 2.5. BET Analysis

Due to the low amount of adsorbed/desorbed nitrogen, the determination of porosity and specific surface area (SSA) for the samples thermally treated at 1000 °C was not possible. The SSA below the method detection limit (0.5 m^2^/g) suggests that all ferrites have a non-porous structure, probably due to particle agglomeration that limits nitrogen absorption.

### 2.6. TEM Analysis

**Figure 4:**
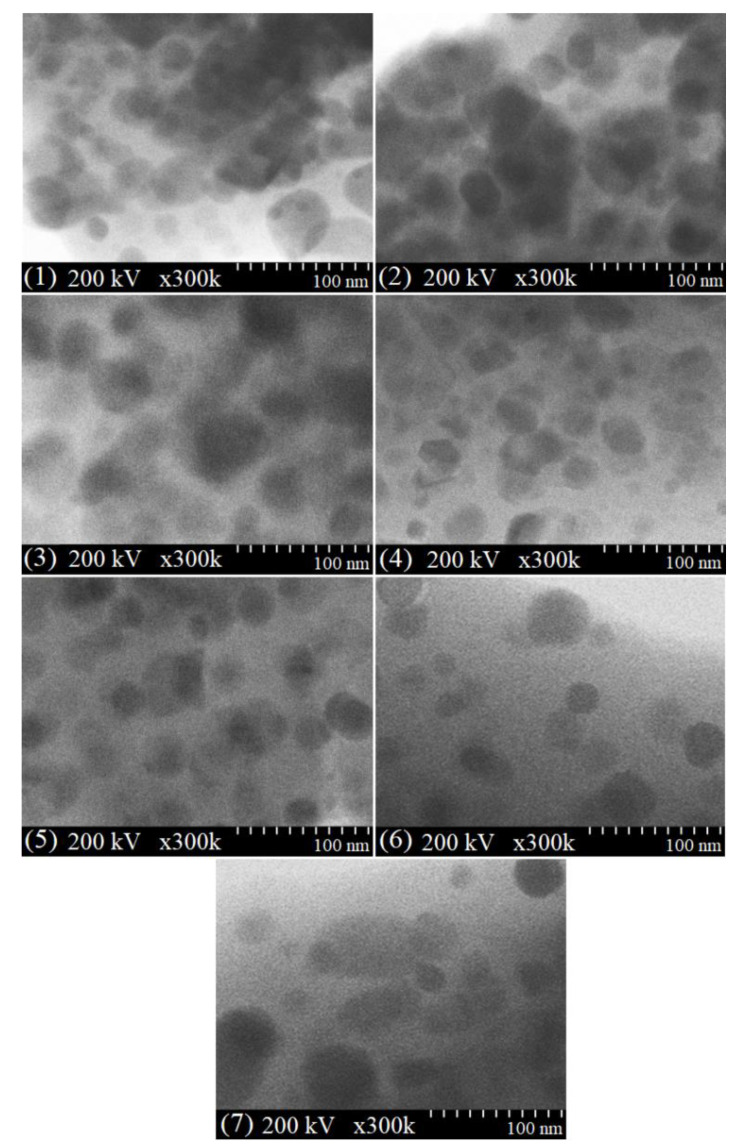
TEM images of Zn_0.6_Co_0.4_Fe_2_O_4_@SiO_2_ (1), Ni_0.1_Zn_0.5_Co_0.4_Fe_2_O_4_@SiO_2_ (2), Ni_0.2_Zn_0.4_Co_0.4_Fe_2_O_4_@SiO_2_ (3), Ni_0.3_Zn_0.3_Co_0.4_Fe_2_O_4_@SiO_2_ (4), Ni_0.4_Zn_0.2_Co_0.4_Fe_2_O_4_@SiO_2_ (5), Ni_0.5_Zn_0.1_Co_0.4_Fe_2_O_4_@SiO_2_ (6) and Ni_0.6_Co_0.4_Fe_2_O_4_@SiO_2_ (7) samples at 1000 °C.

The TEM images of the mixed Ni-Zn-Co ferrites following thermal treatment at 1000 °C (Figure 4) reveal spherical, small (high Zn content), or large (high Ni content) nanoparticles that form large spongy aggregates. 

The formation of agglomerates with irregular morphology composed of high Zn content ferrite particles and the homogenous dispersion of high Ni content ferrite particles is also remarked. The small grains have a high surface area to volume ratio and allow faster oxygen diffusion than the larger grains, leading to an increase in the stoichiometry of the sample [35]. Although the small particles are closely arranged together, a clear boundary between adjacent particles is still observed. The average particle size is 34–36 nm, the difference being attributed to the grain boundary motion that exerts a dragging force, while the pores delay the force over the grain [41]. Moreover, the driving force increases the grain boundaries over the pores, resulting in lower pore volume and higher density [41]. The average crystallite size estimated by XRD is close to the particle size determined by TEM, the slight differences being attributed to the amorphous SiO_2_ matrix and large-size nanoparticles [43,44,50].

### 2.7. UV-VIS Analysis

**Figure 5:**
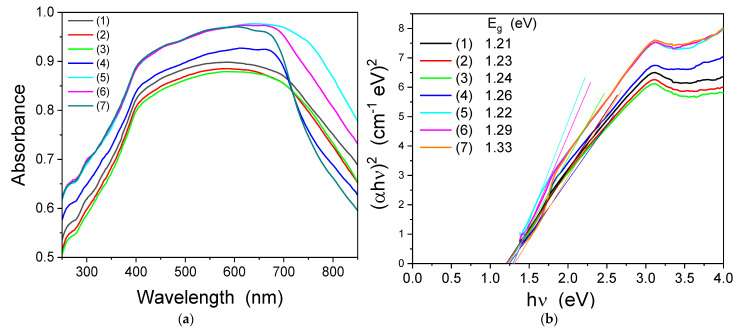
(**a**) UV-Vis absorption spectra and (**b**) Tauc’s plot of the of Zn_0.6_Co_0.4_Fe_2_O_4_@SiO_2_ (1), Ni_0.1_Zn_0.5_Co_0.4_Fe_2_O_4_@SiO_2_ (2), Ni_0.2_Zn_0.4_Co_0.4_Fe_2_O_4_@SiO_2_ (3), Ni_0.3_Zn_0.3_Co_0.4_Fe_2_O_4_@SiO_2_ (4), Ni_0.4_Zn_0.2_Co_0.4_Fe_2_O_4_@SiO_2_ (5), Ni_0.5_Zn_0.1_Co_0.4_Fe_2_O_4_@SiO_2_ (6) and Ni_0.6_Co_0.4_Fe_2_O_4_@SiO_2_ (7) samples at 1000 °C.

The optical response of the samples was evaluated by UV-Vis spectroscopy. The UV-Vis absorption (Figure 5a) shows that all the samples have a broad response in the visible range. Based on the absorption spectra and using the Tauc’s relation [50], the band gap energy of the samples was evaluated (Figure 5b). 

The band gap energy values are in the range 1.21–1.49 eV, are lower than that of NiFe_2_O_4_ (2.2 eV), ZnFe_2_O_4_ (1.91 eV) and CoFe_2_O_4_ (2.31 eV), and are comparable to those reported for CoFe_2_O_4_ xerogel calcined at 500 °C (1.5 eV) [33,50]. The band gap of our samples was also lower than those of Ni_x_Co_1-x_Fe_2_O_4_ (1.37–1.78 eV) obtained by coprecipitation [52]. The optical band gap of the samples is due to the d-d transition. The crystal field splits the d level in the e_g_ and t_2g_ levels and the band gap energy depends on octahedral (B) and tetrahedral (A) sites. The band gap energy, in the case of the octahedral site, is higher than that of the tetrahedral (A) site [53]. The variation of the band gap energy, by replacing Zn^2+^ ions with Ni^2+^, can be explained by the redistribution of Ni^2+^ ions between the octahedral (B) and tetrahedral (A) sites. In the XRD data, the peaks corresponding to the plane (220) and (422) are sensitive to the tetrahedral (A) site, whereas the peak corresponding to the (222) plane is sensitive to the octahedral (B) site [54,55]. The values of the I(220)/I(222) ratio, for the samples annealed at 1000 °C, are 4.29 for Zn_0.6_Co_0.4_Fe_2_O_4_@SiO_2_, 3.79 for Ni_0.1_Zn_0.5_Co_0.4_Fe_2_O_4_@SiO_2_, 4.26 for Ni_0.2_Zn_0.4_Co_0.4_Fe_2_O_4_@SiO_2_, 4.10 for Ni_0.3_Zn_0.3_Co_0.4_Fe_2_O_4_@SiO_2_, 3.52 for Ni_0.4_Zn_0.2_Co_0.4_Fe_2_O_4_@SiO_2_, 3.22 for Ni_0.5_Zn_0.1_Co_0.4_Fe_2_O_4_@SiO_2_ and 3.77 for Ni_0.6_Co_0.4_Fe_2_O_4_@SiO_2_, which indicates that the population at the tetrahedral (A) site tends to decrease with the increase of the Ni^2+^ ions. These findings are correlated with the optical band gap values, which increases with the increase of Ni^2+^ content. The low band gap energy makes our samples suitable for the absorption of visible light. The activation energy of the Co_0.6_Zn_0.4_Ni_x_Fe_2-x_O_4_ ferrite nanoparticles obtained by sol-gel route decreased with the increasing Ni content, from 2.71 eV (x = 0.2) to 1.46 eV (x = 1) [33].

### 2.8. Sonophotocatalytic Activity

**Figure 6:**
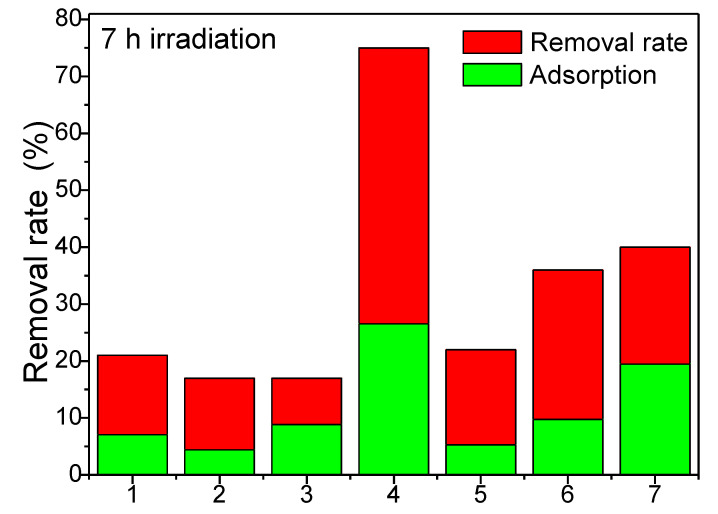
Removal rate of Zn_0.6_Co_0.4_Fe_2_O_4_@SiO_2_ (1), Ni_0.1_Zn_0.5_Co_0.4_Fe_2_O_4_@SiO_2_ (2), Ni_0.2_Zn_0.4_Co_0.4_Fe_2_O_4_@SiO_2_ (3), Ni_0.3_Zn_0.3_Co_0.4_Fe_2_O_4_@SiO_2_ (4), Ni_0.4_Zn_0.2_Co_0.4_Fe_2_O_4_@SiO_2_ (5), Ni_0.5_Zn_0.1_Co_0.4_Fe_2_O_4_@SiO_2_ (6) and Ni_0.6_Co_0.4_Fe_2_O_4_@SiO_2_ (7) samples.

The sonophotocatalytic activity of the samples was evaluated using an RhB synthetic solution under visible irradiation. Before visible irradiation, the samples were kept in the dark for 1 h to reach the adsorption-desorption equilibrium. The adsorption capacity of the sample varied between 7–28%. The adsorption properties depend on the surface sites and specific surface area. In our case, the samples had almost identical particle sizes; thus, those surface sites were responsible for the adsorption properties. The removal rate (Figure 6) was evaluated after 7 h of visible irradiation and varied between 16 and 75%. Similar removal efficiencies (83.9%) for methylene blue were obtained using Ni-Cu-Zn ferrite@SiO_2_@TiO_2_ by Chen et al. [52,55].

The sample with similar Zn^2+^ and Ni^2+^ ions content (Ni_0.3_Zn_0.3_Co_0.4_Fe_2_O_4_@SiO_2_) shows the highest removal capacity, indicating that the equilibrium between Ni-ferrite and Zn-ferrite assures the best photocatalytic performance. In addition, based on the quantitative crystalline phase analysis, this sample contains a lower amount of α-Fe_2_O_3_ (2%) compared with samples Zn_0.6_Co_0.4_Fe_2_O_4_@SiO_2_ (1), Ni_0.1_Zn_0.5_Co_0.4_Fe_2_O_4_@SiO_2_ (2), Ni_0.2_Zn_0.4_Co_0.4_Fe_2_O_4_@SiO_2_ (3), which means that in the case of this sample, α-Fe_2_O_3_ does not significantly influence photocatalytic activity.

**Figure 7:**
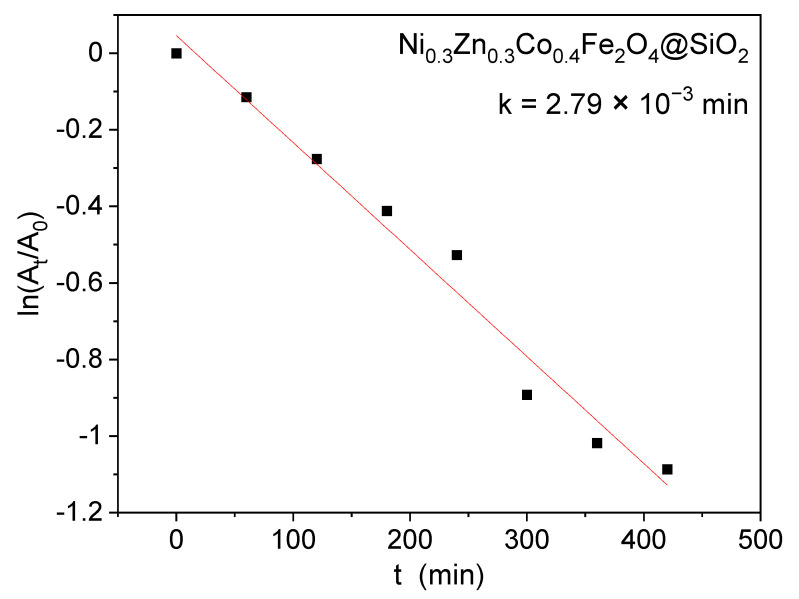
Photodegradation kinetics of RhB in the presence of Ni_0.3_Zn_0.3_Co_0.4_Fe_2_O_4_@SiO_2_.

For this sample, the photodegradation kinetic was analyzed with respect to the absorbance of RhB using the pseudo-first order kinetic model (Equation (6)).

(6) $$-ln\frac{A_{t}}{A_{0}^{*}}=k_{i}\cdot t$$

where A_t_ is the absorbance of RhB at time t, $A_{0}^{*}\;$is the absorbance of RhB at time t_0_ and k_i_ is the apparent kinetic constant (min^−1^). A linear relationship with the irradiation time (Figure 7), with a rate constant of 2.79 × 10^−3^ min was obtained.

## 3. Materials and Methods

### 3.1. Synthesis 

The Ni-Zn-Co ferrite embedded in SiO_2_ matrix (60% wt. ferrite, 40% wt. SiO_2_) were synthesized by sol-gel method using Ni(NO_3_)_2_∙6H_2_O, Zn(NO_3_)_2_∙6H_2_O, Co(NO_3_)_2_∙6H_2_O, Fe(NO_3_)_3_∙9H_2_O, 1,4-butanediol (1,4-BD), tetraethyl orthosilicate (TEOS), ethanol and HNO_3_ 65%, using a Ni:Zn:Co:Fe molar ratio of 0:6:4:20 (Zn_0.6_Co_0.4_Fe_2_O_4_@SiO_2_), 1:5:4:20 (Ni_0.1_Zn_0.5_Co_0.4_Fe_2_O_4_@SiO_2_), 1:2:2:10 (Ni_0.2_Zn_0.4_Co_0.4_Fe_2_O_4_@SiO_2_), 3:3:4:20 (Ni_0.3_Zn_0.3_Co_0.4_Fe_2_O_4_@SiO_2_), 2:1:2:10 (Ni_0.4_Zn_0.2_Co_0.4_Fe_2_O_4_@SiO_2_), 5:1:4:20 (Ni_0.5_Zn_0.1_Co_0.4_Fe_2_O_4_@SiO_2_), 6:0:4:20 (Ni_0.6_Co_0.4_Fe_2_O_4_@SiO_2_) and a nitrate:1,4-BD:TEOS molar ratio of 1:1:0.67. All chemicals were of analytical grade (Merck) and used without further purification. The resulting sols were kept at room temperature until gelation (5 weeks), ground, dried at 40 °C (5 h), and then subjected to thermal treatment 1000 °C.

### 3.2. Characterization 

The formation and decomposition of the carboxylate-type precursor were investigated by thermogravimetry (TG) and differential thermal analysis (DTA) using the SDT Q600 thermogravimeter, in air, up to 1000 °C, at 10 °C·min^−1^ heating rate, using alumina standards. The FT-IR spectra were recorded on KBr pellets containing 1% samples using a Perkin Elmer Spectrum BX II spectrometer, while the XRD patterns were recorded at room temperature using a Bruker D8 Advance diffractometer with CuK_α1_ radiation (λ = 1.54060 Å). The Ni/Zn/Co molar ratios were confirmed using Perkin Elmer ICP-OES Optima 5300 DV (Norwalk, CT, USA) after closed-vessel microwave-assisted aqua regia digestion using a Speedwave Xpert system (Berghof, Germany). The specific surface area (SSA) was obtained using the BET model from N_2_ adsorption-desorption isotherms recorded at −196 °C by a Sorptomatic 1990 (Thermo Fisher Scientific) instrument. The UV–VIS absorption spectra were recorded using a JASCO V570 UV–VIS-NIR spectrophotometer, equipped with a JASCO ARN-475 absolute reflectivity measurement accessory.

### 3.3. Sonophotocatalysis 

The sonophotocatalytic activity of the samples was evaluated against RhB solution under visible light irradiation in a Laboratory-Visible-Reactor system using a 400 W halogen lamp (Osram) and an ultrasonic bath. The catalyst (10 mg) was suspended in an aqueous solution of RhB (1.0 × 10^−5^ mol L^−1^, 20 mL), and the mixture was stirred in the dark to achieve the adsorption equilibrium on the catalyst surface. Each degradation experiment was conducted for 240 min. Samples from a given mixture (3.5 mL) were withdrawn for analysis every 60 min. After separating the catalyst from the suspensions with a permanent magnet, the solution was analyzed using a UV–Vis spectrophotometer by recording the maximum absorbance of RhB at 554 nm. The sonophotocatalytic activity was estimated based on the calculated degradation rate. Before the sonophotodegradation experiments, the RhB adsorption on the surface of the nanoparticles was analyzed. The adsorption was verified in the dark by mixing the photocatalyst into the RhB solution for 60 min until the adsorption-desorption equilibrium was reached.

## 4. Conclusions

Ni-Zn-Co ferrites, with different Ni:Zn:Co ratios (Zn_0.6_Co_0.4_Fe_2_O_4_, Ni_0.1_Zn_0.5_Co_0.4_Fe_2_O_4_, Ni_0.2_Zn_0.4_Co_0.4_Fe_2_O, Ni_0.3_Zn_0.3_Co_0.4_Fe_2_O_4_, Ni_0.4_Zn_0.2_Co_0.4_Fe_2_O_4_, Ni_0.5_Zn_0.1_Co_0.4_Fe_2_O_4_, and Zn_0.6_Co_0.4_Fe_2_O_4_@SiO_2_), embedded in SiO_2_ were obtained by sol-gel method, followed by thermal treatment at 1000 °C. The thermal analysis revealed the formation and decomposition of metal succinate precursors in two stages, with distinct formation and decomposition of divalent (Ni^2+^, Zn^2+^, Co^2+^) and trivalent (Fe^3+^) succinates. The shapes of the DTA curves are similar, with the exception of the divalent metal’s succinate decomposition stage, where for samples with high Ni content, the intensity of the exothermic peak decreases and shifts to higher temperatures. The total mass losses vary between 54.4–58.5%. The precursor formation and their decomposition into ferrites, as well as the formation of the silica matrix, are also confirmed by the FT-IR spectra. The XRD revealed the presence of well-crystallized ferrites along two crystalline phases of the SiO_2_ matrix (cristobalite and tridymite). In samples with high Zn content, traces of hematite were also identified. The agglomeration of particles and the particle size of Ni-Zn-Co ferrites increase with the increasing Ni content, from 34 nm to 40 nm. All of the samples show a good optical response in the visible range, the best sonophotocatalytic performance being found for the Ni_0.3_Zn_0.3_Co_0.4_Fe_2_O_4_@SiO_2_ sample, most likely due to the equilibrium between Ni-ferrite and Zn-ferrite.

## Figures and Tables

**Table 1:** Parameters obtained from TEM, XRD and ICP-OES analysis for samples thermally treated at 1000 °C.

Nanocomposite	D_TEM_ (nm)	D_XRD_ (nm)	D_C_ (%)	a (Å)	V (Å^3^)	L_A_ (Å)	L_B_ (Å)	Quantitative Analysis (%)	Ni/Zn/Co
Zn_0.6_Co_0.4_Fe_2_O_4_@SiO_2_	34	33.2	89.0	8.456	605	14.6	12.0	12% Zn_0.6_Co_0.4_Fe_2_O_4_/ 4% α-Fe_2_O_3_/ 84% SiO_2_	0/0.60/0.39
Ni_0.1_Zn_0.5_Co_0.4_Fe_2_O_4_@SiO_2_	35	33.6	89.4	8.405	594	14.5	11.9	14% Ni_0.1_Zn_0.5_Co_0.4_Fe_2_O_4_/ 4% α-Fe_2_O_3_/ 82% SiO_2_	0.08/0.49/0.39
Ni_0.2_Zn_0.4_Co_0.4_Fe_2_O_4_@SiO_2_	36	34.1	89.8	8.375	587	14.5	11.8	15% Ni_0.2_Zn_0.4_Co_0.4_Fe_2_O_4_/ 4% α-Fe_2_O_3_/ 81% SiO_2_	0.19/0.38/0.41
Ni_0.3_Zn_0.3_Co_0.4_Fe_2_O_4_@SiO_2_	36	34.5	90.1	8.366	586	14.5	11.8	17% Ni_0.3_Zn_0.3_Co_0.4_Fe_2_O_4_/ 2% α-Fe_2_O_3_/ 81% SiO_2_	0.28/0.27/0.42
Ni_0.4_Zn_0.2_Co_0.4_Fe_2_O_4_@SiO_2_	37	35.0	90.4	8.354	583	14.4	11.8	13% Ni_0.4_Zn_0.2_Co_0.4_Fe_2_O_4_/ 87% SiO_2_	0.41/0.08/0.38
Ni_0.5_Zn_0.1_Co_0.4_Fe_2_O_4_@SiO_2_	38	36.6	90.6	8.346	581	14.4	11.8	15% Ni_0.5_Zn_0.1_Co_0.4_Fe_2_O_4_/ 85% SiO_2_	0.49/0.08/0.38
Ni_0.6_Co_0.4_Fe_2_O_4_@SiO_2_	40	36.9	90.8	8.335	579	14.4	11.7	20% Ni_0.6_Co_0.4_Fe_2_O_4_/ 80% SiO_2_	0.61/0.39/0

## Data Availability

Not applicable.
